# Reproductive Capability Is Associated with Lifespan and Cause of Death in Companion Dogs

**DOI:** 10.1371/journal.pone.0061082

**Published:** 2013-04-17

**Authors:** Jessica M. Hoffman, Kate E. Creevy, Daniel E. L. Promislow

**Affiliations:** 1 Department of Genetics, University of Georgia, Athens, Georgia, United States of America; 2 Department of Small Animal Medicine and Surgery, College of Veterinary Medicine, University of Georgia, Athens, Georgia, United States of America; University of Turku, Finland

## Abstract

Reproduction is a risky affair; a lifespan cost of maintaining reproductive capability, and of reproduction itself, has been demonstrated in a wide range of animal species. However, little is understood about the mechanisms underlying this relationship. Most cost-of-reproduction studies simply ask how reproduction influences age at death, but are blind to the subjects' actual causes of death. Lifespan is a composite variable of myriad causes of death and it has not been clear whether the consequences of reproduction or of reproductive capability influence all causes of death equally. To address this gap in understanding, we compared causes of death among over 40,000 sterilized and reproductively intact domestic dogs, *Canis lupus familiaris*. We found that sterilization was strongly associated with an increase in lifespan, and while it decreased risk of death from some causes, such as infectious disease, it actually increased risk of death from others, such as cancer. These findings suggest that to understand how reproduction affects lifespan, a shift in research focus is needed. Beyond the impact of reproduction on *when* individuals die, we must investigate its impact on *why* individuals die, and subsequently must identify the mechanisms by which these causes of death are influenced by the physiology associated with reproductive capability. Such an approach may also clarify the effects of reproduction on lifespan in people.

## Introduction

Models for life history evolution assume that investment in reproduction comes at the cost of survival. Numerous studies in nematodes, fruit flies and mice have found that reproduction often–but not always–shortens lifespan [1], [2], [3], [4], and scientists disagree over whether reproduction in humans increases, decreases, or has no effect on lifespan (e.g., [5], [6], [7], but see [8]). We submit that these inconsistent results are due to the fact that these studies examine the effect of reproduction on *when* individuals die, but not on *why* they die. Surprisingly, there currently are no comprehensive studies on the specific causes of mortality associated with reproductive capability or sterilization status. While invertebrate species such as *Caenorhabditis elegans* and *Drosophila melanogaster* serve as powerful model systems for genetic and molecular investigations, we know little about actual causes of mortality in these species. Studies on worms and flies are unlikely to explain whether reproduction itself and the physiology associated with reproductive capability affect all causes of mortality, or only certain ones. To address this question, we need a model system that is not only well characterized genetically, but is equally well characterized medically, so that we can investigate the underlying disease states that lead to mortality.

The domestic dog exhibits dramatic breed-associated phenotypic variation not only in morphology and behavior [9], but also in causes of death [10]. Additionally, elective surgical sterilization by ovariohysterectomy ("spay") or orchiectomy ("castration" or "neuter") is commonly performed at a young age in pet dogs in North America for the management and behavioral benefits it confers [11]. By electing whether or not to sterilize their dogs, dog owners have inadvertently carried out a large-scale epidemiologic experiment on the consequences of effectively eliminating reproductive capability. Previous studies in dogs have examined the effects of sterilization status on a variety of specific diseases. For example, sterilized dogs generally show an increase in rates of specific cancers [12], [13], [14], [15] with the exception of mammary cancer, which is relatively rare in sterilized dogs [16]. However, in all but one of these studies [15], the relationship between sterilization and disease-specific risk of death is confounded with age. If elective sterilization increases life expectancy, then sterilized dogs might have a higher occurrence of diseases that occur late in life (such as cancer) simply because sterilized dogs live longer.

Here we determined the effects of sterilization not only on longevity, but also on the pathophysiological causes of death, controlling for the confounding effects of age. We examined causes of death in over 40,000 domestic dogs that died in veterinary teaching hospitals from 1984 to 2004. By comparing causes of death in dogs that had undergone elective surgical sterilization and those that had not, we were able to measure the lifespan cost of maintaining reproductive capability, and to determine the categories of disease associated with this cost.

## Materials and Methods

### Data Collection

The Veterinary Medical Data Base (VMDB, http://www.vmdb.org) contains abstracted medical records of animals presented to North American veterinary teaching hospitals since 1964. Each animal's record includes species, sex, sterilization status, age class, weight class, breed, and diagnoses made during the visit. We obtained the VMDB records for all dogs whose hospital visits resulted in death between the years 1984–2004, including all diagnoses recorded at the time of death. Dogs under one year of age or with unknown sterilization status were removed from analyses. Records of dogs meeting these criteria (Full Cohort, FC) were used to determine lifespan, and for assessment of all diagnoses present at the time of death.

For each dog, a single diagnosis was identified as the cause of death and categorized into one of nine pathophysiologic processes (PP; congenital, degenerative, infectious, immune-mediated, metabolic, neoplastic, toxic, traumatic and vascular) as previously described [10]. Some diagnoses contained insufficient information to allow PP categorization and these records were excluded from cause of death analysis. Congenital causes of death were also removed from subsequent analysis because they would have been present before the time that sterilization was or was not elected. Records remaining after these exclusions (Cause of Death Cohort, CODC) were used for cause of death analysis.

### Lifespan

In the VMDB, dogs are classified into nine age bins (bins evaluated: bin 4∶1–2 years; bin 5∶2–4 years; bin 6∶4–7 years; bin 7∶7–10 years; bin 8∶10–15 years; and bin 9∶15 years and older). Age at death was assigned as the midpoint of each bin for all dogs in that bin, and for bin 9, the age assigned was 17.5 years. All analyses were performed using the software program R [17]. To determine the effect of sterilization on survival, we applied log-rank tests and generated Kaplan Meier plots using the R package “survival” [18] for FC dogs in the dataset, analyzing males and females separately.

### Cause of Death

If sterilized dogs live longer and if the frequency of a particular cause of death increases with age, then it might appear that sterilization causes an increase in a particular cause of death, when it simply changes the age-distribution of death. To correct for this potential confound, we analyzed causes of death in the CODC dogs stratified by age using a Cochran-Mantel-Haenzel (CMH) test [19], which provides a stratified chi-squared test with one degree of freedom. Pooled odds ratios and 95% confidence intervals were calculated with intact dogs as the reference. We also evaluated differences between sterilized and intact dogs for each PP categorical cause of death within each age bin using a chi-squared test.

Different causes of death in dogs are more prevalent at different ages, so we ran a cumulative incidence model to determine the effects of age on the risk of a specific cause of death and to identify differences in these age-related effects between sterilized and intact dogs. We implemented a cumulative incidence model based on competing risks data using the “cmprsk” package in R [20].

Using the FC dogs, discretely defined infectious and neoplastic diagnoses that were present in more than 1% of the population at the time of death, regardless of cause of death, were also analyzed. To test for effects of sterilization within each diagnosis controlling for age, we used the CMH test described above.

Previous studies have shown that breeds differ both by rates and by causes of mortality [10]. To ensure that patterns observed in the CMH test were not confounded by differences among breeds, we carried out two additional analyses. First, we performed a logistic regression for each PP categorical cause of death using age and sterilization status as fixed effects and breed as a random effect using the “MASS” package in R [21]. Second, for each PP in which a pattern of sterilization-associated differences in frequency was detected, CMH tests were also run separately for each of the 24 most frequently encountered breeds within the dataset (mixed breed dogs were considered a single group). This allowed us to determine if sterilization had varying effects among breeds. Dog breed sizes were classified by the average of adult male and female body weights reported in breed standards, or compiled from veterinary and public resources for those breeds whose standard does not include a weight. We used the size categories small (up to 10 kg), medium (10.1–25 kg), large (25.1–40 kg), and giant (>40 kg).

## Results

### Full Cohort

The initial dataset contained 80,958 records of dog death. When juvenile dogs and those with unknown sterilization status were removed there were 70,574 FC dogs, representing 185 breeds. The average number of diagnoses recorded per dog was 2.9 (range 1–32). Overall, 30,770 (43.6%) dogs were intact and 39,804 (56.4%) dogs were sterilized at the time of death. The mean age of death for intact dogs was 7.9 years versus 9.4 years for sterilized dogs.

### Cause of Death Cohort

We were able to identify the PP category for the specific cause of death in 41,045 dogs, and we removed 906 dogs whose cause of death was congenital (occurring primarily in the earliest of the age bins that we analyzed here). This enabled the inclusion of 40,139 dogs in the CODC for analysis of the relationship between sterilization and cause of death.

### Lifespan

**Figure 1:**
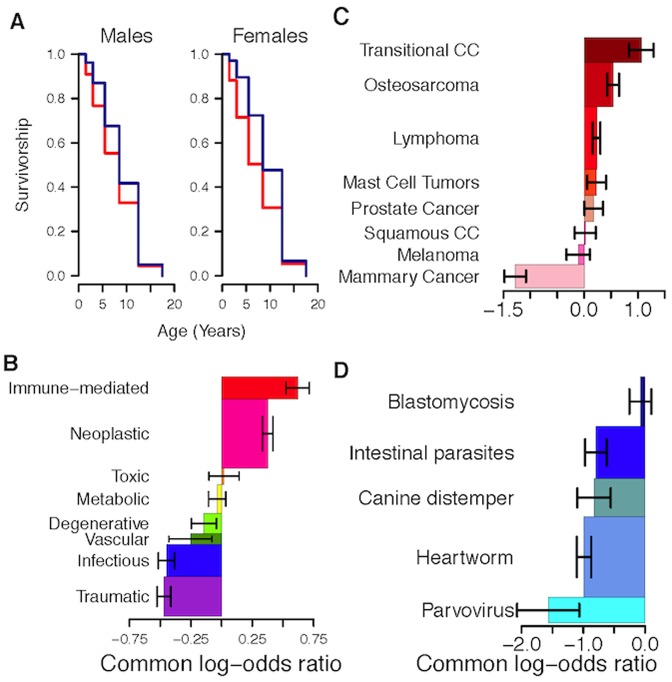
Effect of sterilization on longevity and diagnoses. (A) Kaplan-Meier plots of longevity for males (left) and females (right). Blue lines indicate sterilized dogs and red lines indicate intact dogs. (B) Common log-odds ratios with 95% confidence intervals (CI) for pathophysiological processes (PP). Height of each bar indicates the relative frequency of each PP among all deaths. (C) Effects of sterilization on specific neoplastic diagnoses, showing common log odds ratios and 95% CI. Height of each bar indicates fraction of individuals with this diagnosis at the time of death. Transitional CC – transitional cell carcinoma; Squamous CC – squamous cell carcinoma. All cancers significant at *P*<0.01 except prostate cancer, squamous cell carcinoma and melanoma (*P*>0.05). (D) As in Figure 1C, but for specific infectious disease diagnoses. All infectious diseases significant at *P*<0.01 except blastomycosis (*P*>0.4).

We found that sterilization significantly affected survival in both males (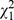 = 446, *P*<10^−6^) and females (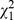 = 1372, *P*<10^−6^) (Figure 1A). Sterilization increased life expectancy by 13.8% in males and 26.3% in females among the FC dogs.

### Cause of Death

We found a striking effect of sterilization on cause of death (Figure 1B). Sterilized dogs were dramatically less likely to die of infectious disease (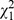 = 184.4, *P*<10^−6^), trauma (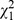 = 268.7, *P*<10^−6^), vascular disease (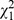 ^= ^8.25, *P* = 0.004), and degenerative disease (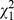 = 7.7, *P* = 0.006). In contrast, sterilized dogs died more commonly from neoplasia (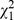 = 300.4, *P*<10^−6^) and immune-mediated disease (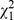 = 167.2, *P*<10^−6^). We saw effects of sterilization both on common causes of death, as well as on more rare causes (e.g., vascular disease). We also found that even within specific age bins, there were visible differences in causes of death for sterilized and intact dogs (Figure S1).

We expected that the consequences of sterilization would differ significantly between the sexes, as the endocrinological consequences of sterilization should differ between males and females. Surprisingly, both the direction and magnitude of the effect of sterilization on cause of death was markedly similar in males and females (Table 1). Results from the logistic regression, controlling for the effects of breed as well as age, mirrored the initial findings (Table S1).

**Table 1:** Risk of death by pathophysiologic process (PP) for sterilized dogs.

Sterilized males					
Process	Odds-ratio	Lower	Upper	Chi-sq value	P-value
Traumatic	−0.46	−0.54	−0.37	122.92	<2.2e−16
Infectious	−0.46	−0.56	−0.37	89.48	<2.2e−16
Vascular	−0.35	−0.61	−0.08	6.24	0.01
Degenerative	−0.15	−0.29	0.00	3.75	0.05
Metabolic	−0.09	−0.19	0.01	3.11	0.08
Toxic	0.04	−0.14	0.22	0.17	0.68
Neoplastic	0.43	0.37	0.49	194.26	<2.2e−16
Immune-Mediated	0.51	0.37	0.65	52.22	<2.2e−16
**Sterilized females**					
**Process**	**Odds-ratio**	**Lower 95% CI**	**Upper 95% CI**	**Chi-sq value**	**P-value**
Traumatic	−0.44	−0.53	−0.35	101.61	<2.2e−16
Infectious	−0.49	−0.59	−0.39	101.74	<2.2e−16
Vascular	−0.31	−0.56	−0.06	5.61	0.02
Degenerative	−0.20	−0.35	−0.04	6.10	0.01
Metabolic	0.00	−0.11	0.11	0.00	0.99
Toxic	−0.10	−0.28	0.08	1.15	0.28
Neoplastic	0.42	0.35	0.49	152.27	<2.2e−16
Immune-Mediated	0.54	0.39	0.68	56.32	<2.2e−16

Odds ratios for each of the eight PP are shown. Data for sterilized males (top) and sterilized females (bottom) are shown separately, and intact dogs of the same sex served as the reference population for each group.

Because causes of death vary with age, we ran a cumulative incidence analysis to determine if the pathophysiological risks of death associated with sterilization status differ among age-classes (Figure S2). Most differences in causes of death between sterilized and intact dogs (intact dogs at greater risk for infectious and traumatic causes of death, sterilized dogs at greater risk for neoplastic and immune-mediated causes of death) remain significantly different under a cumulative incidence model using competing risk data (Table S2). However, while our CMH tests show that degenerative causes of death are significantly more frequent in intact dogs, the cumulative incidence model fails to find a significant difference between intact and sterilized dogs for this PP category.

### Diagnoses at the Time of Death

Given the prevalence of neoplasia and infectious disease in these dogs and the relevance of those diseases to humans, within these categories we further examined specific diagnoses that were present in more than 1% of the FC dog population in a specific age class at the time of death, regardless of cause of death. Eight cancers met these criteria, five of which are among the top ten human cancer diagnoses in the US, according to the SEER Database [22]. Seven of the eight analyzed had higher or unchanged frequency in sterilized dogs; only mammary cancer showed a significantly lower prevalence (Figure 1C). Of five infectious diseases we considered, four had significantly lower frequencies in sterilized dogs (Figure 1D).

### Within-breed Cause of Death

**Figure 2:**
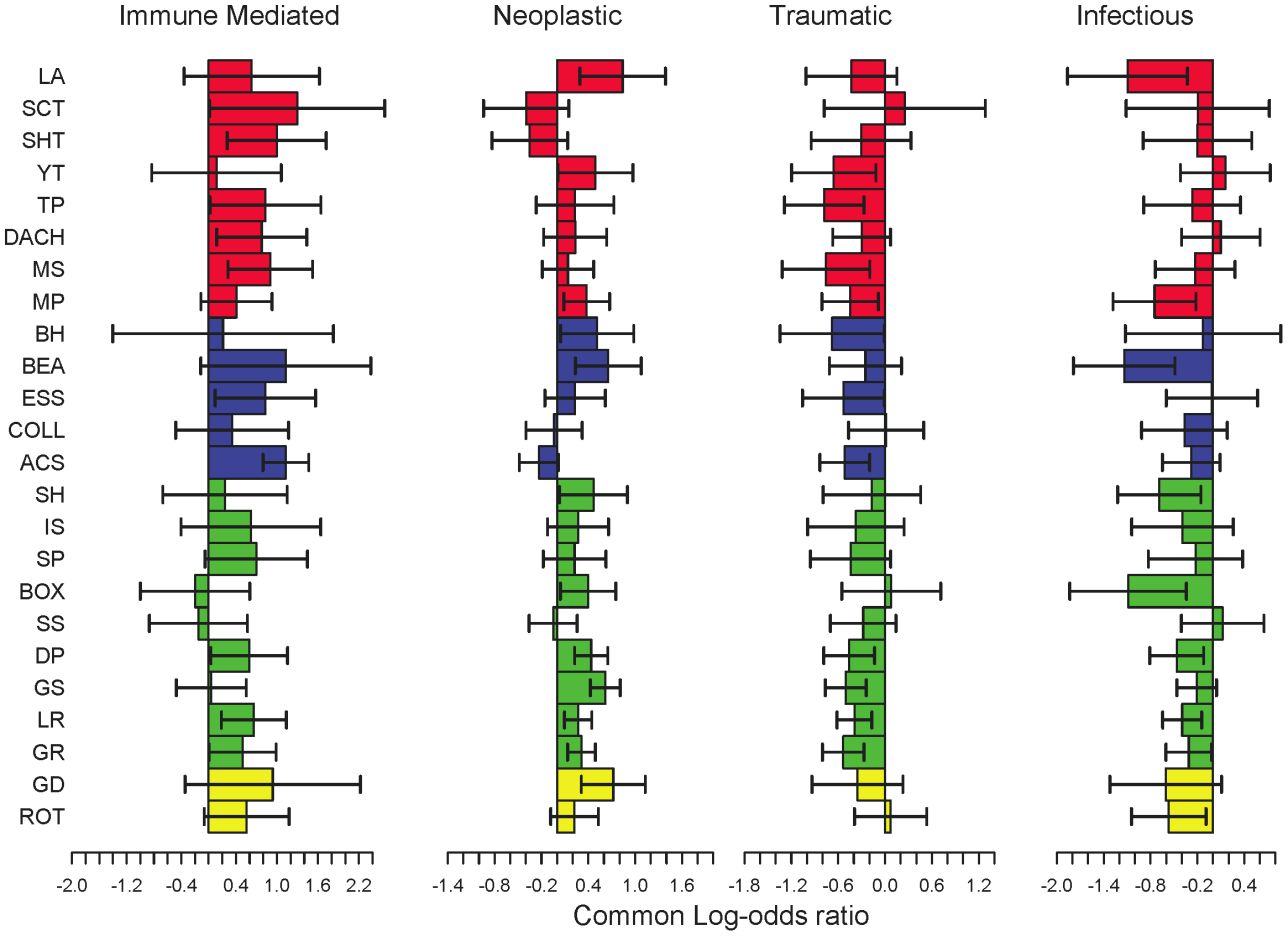
Breed specific causes of mortality. Effects of sterilization on the 24 pure breeds that appear most frequently in our dataset (minimum sample size = 319, median sample size = 517). Bars indicate 95% CI. Colors of odds ratio bars indicate size of the breed (red-small [up to 10 kg], blue-medium [10.1–25 kg], green-large [25.1–40 kg], yellow-giant [>40 kg]). Breed abbreviations are shown (LA: Lhasa Apso, SCT: Scottish Terrier, SHT: Shih Tzu, YT: Yorkshire Terrier, TP: Toy Poodle, DACH: Dachshund, MS: Miniature Schnauzer, MP: Miniature Poodle, BH: Basset Hound, BEA: Beagle, ESS: English Springer Spaniel, COLL: Collie, ACS: American Cocker Spaniel, SH: Siberian Husky, IS: Irish Setter, SP: Standard Poodle, BOX: Boxer, SS: Shetland Sheepdog, DP: Doberman Pinscher, GS: German Shepherd, LR: Labrador Retriever, GR: Golden Retriever, GD: Great Dane, ROT: Rottweiler).

Finally, for the four PP causes of death most affected by sterilization status, the patterns that we observed were recapitulated within individual breeds (Figure 2). Since small-breed dogs are known to have longer lifespans than large-breed dogs [23], [24], we divided the 24 most frequently observed breeds into four size classes (small, medium, large and giant). Despite the longer lifespans seen in small compared to large dogs, the effect of sterilization was relatively consistent among size classes (ANOVA, *P*>0.11 for all processes). Notably, even though death due to neoplasia is relatively rare in the smallest size classes [10], sterilization still increased the risk of neoplasia within these breeds.

## Discussion

Despite a rich literature on the relationship between reproduction and lifespan [1], [2], [25], surprisingly little is understood about the mechanisms by which investment in reproduction affects cause of death. Our analysis of causes of death associated with reproductive capability suggests that further and more detailed studies of reproduction and mortality in companion dogs could shed considerable light on this problem. Companion dogs are an established medical model for humans because the two species experience many of the same spontaneously-occurring diseases, participate in analogous high-caliber medical and surgical care, respond similarly to therapy, and share a daily environment [10], [26], [27], [28], [29]. No other species is similarly able to mirror the human experience of the impacts of environment, lifestyle choices, and medical care on health. Furthermore, in North America 50–75% of pet dogs are electively surgically sterilized, as recommended by the American and Canadian Veterinary Medical Associations, the American Society for the Prevention of Cruelty to Animals, and the Humane Society of the United States. The existence of large numbers of reproductively intact and electively sterilized companion dogs provides an unparalleled opportunity to evaluate outcome differences between the groups.

In our study on companion dogs, we identified many underlying causes of death that shape the composite trait of lifespan. In our study overall, lifespan was greater in the sterilized dogs compared with the reproductively intact dogs. While intact reproductive capability was associated with decreased lifespan in dogs, some causes of death were less frequent in intact dogs. Interestingly, we observed the largest–and opposite–effects in two of the most common causes of death among dogs within our dataset: neoplasia and infectious disease. It is not within the scope of our study to determine the causes of these associations. While the absence of gonadal hormones is an obvious physiological outcome of surgical sterilization, downstream consequences of the absence of gonadal hormones, including altered feedback on pituitary or adrenal hormonal axes, or changes in patterns of growth, development, or behavior may also be significant factors.

Sterilization increased the risk of death due to neoplasia, but did not increase risk for all specific kinds of cancer. Female dogs sterilized before sexual maturity are unlikely to develop mammary cancer because of the decrease in cumulative estrogen exposure associated with the absence of the estrus cycle [30]. However, it is not clear why the frequency of some cancers outside the reproductive system, including lymphoma and osteosarcoma, is influenced by sterilization, while the frequency of others, such as melanoma and squamous cell carcinoma, is not. The increased risk of death due to cancer observed in sterilized dogs could be due to the fact that in both sexes, dogs sterilized before the onset of puberty grow taller than their intact counterparts [31] as a result of reduced estrogen signaling [32]. Recent studies in humans suggest that growth is a risk factor for a number of different cancers [33].

Conversely, sterilized dogs had a decreased risk of death due to infection, and avoidance of infection may partly explain their longer lifespans. The relationship between sterilization and infectious disease could arise due to increased levels of progesterone and testosterone [34] in intact dogs, both of which can be immunosuppressive [35], [36]. Studies in humans, mice and rats reveal patterns of infectious disease morbidity and mortality associated with testosterone and estrogen exposure. However, these patterns vary with host species, type of pathogen, and chronicity of infection [37]. Additionally, sterilization and disease risk might both be correlated with specific canine behaviors. Given the opportunity, intact male dogs are more likely than sterilized dogs to roam, and to fight with other dogs, and intact female dogs show more dominance aggression than spayed females [38], [39]. These behaviors might increase the risks of both infectious and traumatic causes of death among intact dogs.

Limited previous studies on the effects of gonadectomy in humans have found some results consistent with ours. Studies in two different populations have shown that eunuchs live longer than their intact male counterparts [40], [41]. Interestingly, Hamilton and Gordon [40] found that the largest factor influencing the survival difference was the high rate of death due to infections among the intact men. However, the study failed to find differences between the two groups in death due to cancer or trauma, both categories in which sterilization was associated with a large effect in our dataset.

Retrospective studies such as this are not without potential weaknesses. For example, elective surgical sterilization and subsequent veterinary care are potentially associated with socioeconomic status. Owners who cannot afford the cost of sterilization might also lack the resources to provide medical care for diseases that later occur, which might result in sterilized dogs who have access to better medical care appearing to live longer. This issue is unlikely to exert a significant impact on our results, however, as all dogs within our dataset were seen at referral institutions, where costs of care are high. Since dogs in our study were owned by people who could afford the cost of referral from their local veterinary practices to specialty hospitals when their pets became ill, it is unlikely that financial resources were a limiting factor in preventive health management choices such as sterilization [42].

A second potential bias is introduced by the fact that our dataset does not provide the age at which each dog was sterilized, the number of times that intact dogs reproduced, or whether sterilized dogs reproduced prior to sterilization. We cannot extrapolate this information from prior work because while most North American veterinary practitioners currently recommend sterilization at 6–9 months of age for pet dogs, and specifically before the first heat cycle in females [11], there is no large study which reports the actual age at which most dogs are sterilized. If the proportion of dogs becoming sterilized were constant within each age bin, then sterilized dogs could appear to live longer simply because the sterilized group steadily expands with increasing age. Previous research has shown that using gonadectomy as a time-dependent variable can give different estimates of the effect of sterilization on longevity than using sterilization status as a straight yes/no response, and that right-censored lifespans from retrospective studies underestimate population lifespan [43], [44]. However, it was not our objective to define the precise life expectancy for any category of dog, merely the difference between two groups that varied only by sterilization status. Both sterilized and intact dogs were subject to the same limitation (i.e., enrollment at the time of death), and the impact on the groups is expected to be proportionate. Furthermore, when causes of death are compared between reproductively intact and sterilized dogs within age bins, the differential effects of sterilization persist. Thus, even in the youngest dogs, when the consequences of sterilization would have manifested over a shorter period of time, an impact is apparent (Figure S1). Regarding parity, we cannot state that all individuals within the sterilized group were nulliparous, nor that all individuals within the intact group had reproduced. Thus, it is likely that there is some crossover between groups with respect to actual reproductive experience. The effect of this crossover, however, would be to minimize any differences identified between groups; thus, the ability to identify a marked difference in lifespan and cause of death risk in the face of imperfect separation of lifetime reproductive experience substantiates the significance of the effect. We also note that parallel patterns of pathophysiologic process morbidity were evident within all age groups, suggesting that these effects are robust to variation arising from differences in parity within and between groups. Nonetheless, future studies would obviously benefit from data on parity in individual dogs.

Finally, as previously mentioned, the link between sterilization and the observed outcomes cannot currently be known. A direct cause-and-effect relationship between reproduction and cause of death is possible, but the actual relationship is likely more complex. In mammals, removal of gonadal hormones has been shown to alter hematological and coagulation parameters, the pituitary-adrenal axis, satiety, neurotransmitters, thymic tissue, and behavior [11], [45], [46], [47], [48], [49], [50]. Any or all of these factors could mediate the differential causes of death observed between the reproductively intact and sterilized dogs of this report. Documentation of these outcome differences now creates the exciting opportunity to investigate the possible causal mechanisms in dogs and other species.

Although a retrospective, epidemiological study such as this cannot prove causality, our results suggest that close scrutiny of specific causes of death, rather than lifespan alone, will greatly improve our understanding of the cumulative impact of reproductive capability on mortality. Our results strongly demonstrate the need to determine the physiologic consequences of sterilization that influence causes of death and lifespan. Shifting the focus from when death occurs to why death occurs could also help to explain contradictory findings from human studies [e.g., 8].

## Supporting Information

Figure S1 **Differences in cause of death for sterilized and intact dogs.** Pathophysiological plots by age for sterilized (blue circles) and intact (red triangles) dogs. Error bars indicate +/−1 SE. Black asterisks above each age bin indicate significant difference between sterilized and intact dogs for that age bin using a Chi-squared test. P value <0.05.
(TIFF)

Figure S2 **Competing risks plot for the four most significantly different causes of death between sterilized and intact dogs.** Solid lines represent intact dogs, and dashed lines represent sterilized dogs. Each color represents a different cause of death: orange-neoplastic, red-traumatic, black-infectious, blue-immune-mediated.
(TIFF)

Table S1 **Mixed-effect model of the effects of sterilization on cause of death.** Shown are the results for sterilization under each cause of death. The model includes sterilization and age as fixed effects and breed as a random effect.
(DOCX)

Table S2 **Competing risks analysis for each cause of death.** Results indicate significant differences in risk for sterilized and intact dogs, with d.f. = 1 in each case.
(DOCX)
