# Supplementary figures and images for: Reproductive Capability Is Associated with Lifespan and Cause of Death in Companion Dogs
Source: PLoS One. 2013 Apr 17;8(4):e61082. doi: 10.1371/journal.pone.0061082 (PMC3629191; doi:10.1371/journal.pone.0061082)

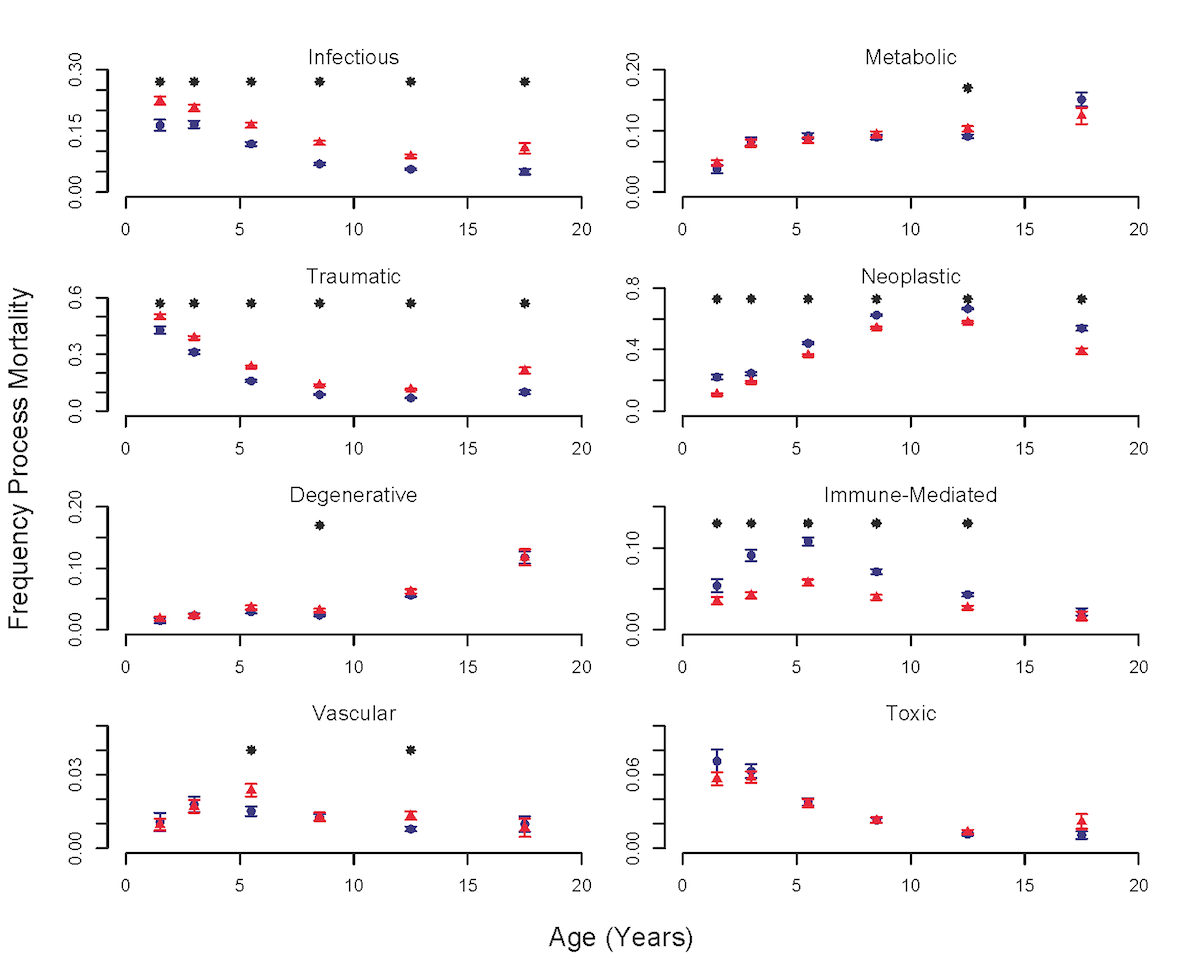

Supplement: Figure S1 — Differences in cause of death for sterilized and intact dogs. Pathophysiological plots by age for sterilized (blue circles) and intact (red triangles) dogs. Error bars indicate +/−1 SE. Black asterisks above each age bin indicate significant difference between sterilized and intact dogs for that age bin using a Chi-squared test. P value <0.05. (TIFF) [file pone.0061082.s001.tiff]

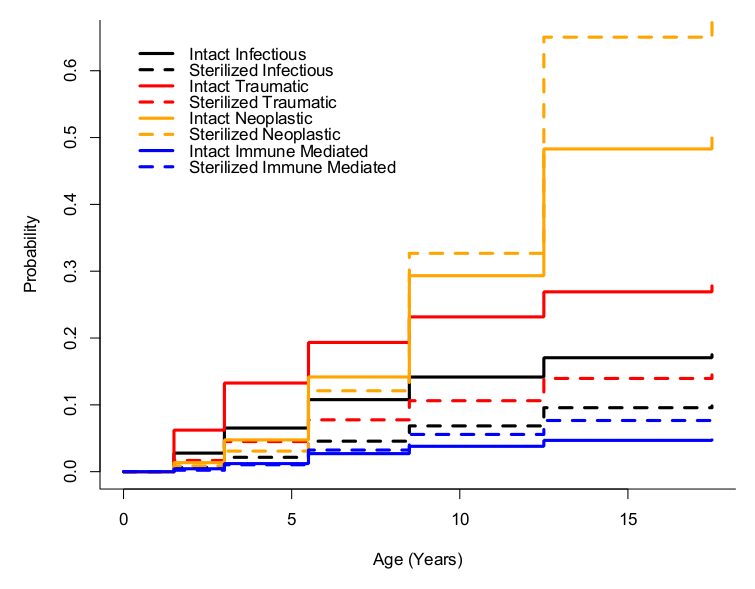

Supplement: Figure S2 — Competing risks plot for the four most significantly different causes of death between sterilized and intact dogs. Solid lines represent intact dogs, and dashed lines represent sterilized dogs. Each color represents a different cause of death: orange-neoplastic, red-traumatic, black-infectious, blue-immune-mediated. (TIFF) [file pone.0061082.s002.tiff]
