# Supplementary material for: Reproductive Capability Is Associated with Lifespan and Cause of Death in Companion Dogs
Source: PLoS One. 2013 Apr 17;8(4):e61082. doi: 10.1371/journal.pone.0061082 (PMC3629191; doi:10.1371/journal.pone.0061082)
Supplement: Table S1 — Mixed-effect model of the effects of sterilization on cause of death. Shown are the results for sterilization under each cause of death. The model includes sterilization and age as fixed effects and breed as a random effect. (DOCX) [file pone.0061082.s003.docx]

| Process | Slope | t-statistic | P value |
| --- | --- | --- | --- |
| Infectious | -0.433 | 12.875 | < 1E-16 |
| Metabolic | -0.013 | -0.369 | 0.713 |
| Traumatic | -0.518 | -17.420 | < 1E-16 |
| Neoplastic | 0.426 | 18.959 | < 1E-16 |
| Degenerative | -0.130 | -2.529 | 0.012 |
| Immune-Mediated | 0.620 | 12.547 | < 1E-16 |
| Vascular | -0.226 | -2.553 | 0.011 |
| Toxic | 0.001 | 0.020 | 0.984 |
