# Supplementary material for: Reproductive Capability Is Associated with Lifespan and Cause of Death in Companion Dogs
Source: PLoS One. 2013 Apr 17;8(4):e61082. doi: 10.1371/journal.pone.0061082 (PMC3629191; doi:10.1371/journal.pone.0061082)
Supplement: Table S2 — Competing risks analysis for each cause of death. Results indicate significant differences in risk for sterilized and intact dogs, with d.f. = 1 in each case. (DOCX) [file pone.0061082.s004.docx]

| Process | Statistic | P-value |
| --- | --- | --- |
| Infectious | 431.291 | < 1E-16 |
| Metabolic | 0.311 | 0.577 |
| Traumatic | 936.642 | < 1E-16 |
| Neoplastic | 776.285 | < 1E-16 |
| Degenerative | 0.011 | 0.917 |
| Immune-Mediated | 126.587 | < 1E-16 |
| Vascular | 13.049 | 3.04E-04 |
| Toxic | 18.570 | 1.64E-05 |
